# Donor Cell Myeloid Sarcoma in an Umbilical Cord Transplant Patient: A Case Report and a Review of the Literature

**DOI:** 10.1155/2015/186869

**Published:** 2015-12-28

**Authors:** Christi Hayes, Bruce Petersen, Adriana Malone

**Affiliations:** ^1^Department of Hematology, Norris Cotton Cancer Center, Dartmouth-Hitchcock Medical Center, Lebanon, NH 03756, USA; ^2^Department of Pathology, Mount Sinai Medical Center, New York, NY 10029, USA; ^3^Bone Marrow and Stem Cell Transplantation, Icahn School of Medicine at Mount Sinai, New York, NY, USA

## Abstract

Donor cell leukemia (DCL) represents a rare complication of allogeneic transplantation. The precise incidence remains unclear, though it may be higher following umbilical cord blood transplants. Here, we present an unusual case of a patient with B-ALL who presented with a donor derived myeloid sarcoma of the heart following a double cord blood transplant. To our knowledge, it is the first case of sarcomatous or chloromatous presentation of DCL following a UCBT.

## 1. Introduction

Donor cell leukemia (DCL) represents a rare complication of allogeneic transplantation. It was first recognized as a distinct entity in 1971 by Fialkow et al. [1] when a 16-year-old female patient with acute lymphoblastic leukemia (ALL) relapsed 62 days after a transplant from her HLA-identical brother. Cytogenetics at relapse revealed only XY metaphases, suggesting that the leukemic cells were donor derived. Sporadic cases have been reported in the literature since that time; however, the precise incidence remains unclear. A large review by European Group for Blood and Marrow Transplantation (EBMT) found 14 cases out of a total of 10,489 allogeneic transplants, for an approximate incidence of 0.1% [2].

The first two cases of donor cell leukemia after umbilical cord blood transplant (UCBT) were reported, more recently, in 2005 [3, 4]. Again, the precise incidence is unknown, but there is some preliminary data to suggest it may be higher than that associated with other stem cell sources. A review of the Tokyo Cord Blood Bank found 4 cases out of a total of 478 transplants, with an incidence close to 1% [5].

Here, we present an unusual case of a patient with prior history of B acute lymphoblastic leukemia (B-ALL) who presented with a donor derived myeloid sarcoma of the pericardium following a double cord blood transplant. To our knowledge, this is the first case of a sarcomatous or chloromatous presentation of DCL following UCBT.

## 2. Case

A 41-year-old man was admitted to our hospital 567 days after a double UCBT for B-ALL with t(1; 19). His treatment history in brief is as follows. After cycle 3A of Hyper-CVAD, he developed symptoms concerning relapse. A bone marrow biopsy confirmed B-ALL. His regimen was changed to Augmented Hyper-CVAD. A repeat bone marrow biopsy was negative for disease.

Given his relapse during treatment, the patient was referred for bone marrow transplant (BMT) evaluation. He was HLA typed and had neither a sibling nor a suitable volunteer unrelated donor match. Thus, he underwent a double UCBT. He received reduced-intensity conditioning prior to his UCBT with cyclophosphamide 50 mg/kg, fludarabine 200 mg/m^2^, and 200 cGy TBI (Cy/Flu/TBI). GVHD prophylaxis included CSA and MMF. After transplant, bone marrow (BM) and peripheral blood (PB) chimerism was 100% donor.

His posttransplant course was complicated by graft-versus-host-disease (GVHD) of skin and the gastrointestinal tract. The GVHD was managed with a brief course of steroids and cyclosporine. Bone marrow and peripheral blood chimerism was 100% donor.

At approximately day 515 after transplant he was noted to be thrombocytopenic and 8% blasts were reported on peripheral blood. A bone marrow biopsy was performed, which revealed a donor derived acute myeloid leukemia (AML), with 22% blasts showing an immature myeloid phenotype by flow cytometry (CD13+, CD34+, HLA-DR+, CD4dim+, CD64dim+, and CD33 partial dim+), negative for CD10 and CD19, both of which had been expressed in the prior B-ALL. His peripheral blood and bone marrow chimerism remained 100% donor. FISH was negative for t(1; 19). He received 1 cycle of decitabine at 20 mg/m^2^ for 10 days and responded well to the treatment. A bone marrow biopsy performed 8 weeks after the completion of the cycle revealed no evidence of AML. Additional cycles of treatments were held secondary to infectious complications.

**Figure 1:**
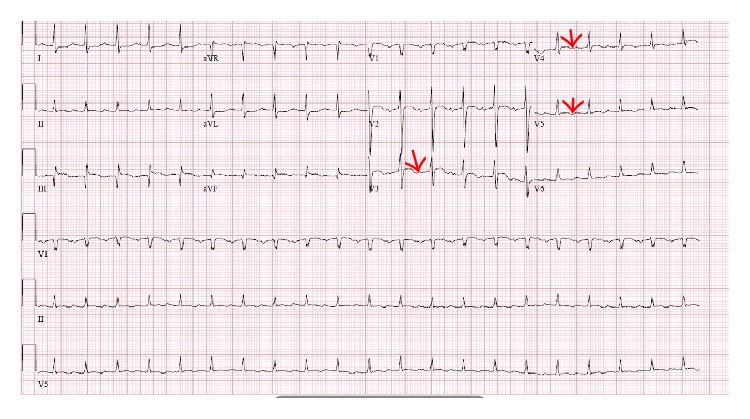
EKG with T-wave inversions in the lateral leads V3–V5 as denoted by arrows.

**Figure 2:**
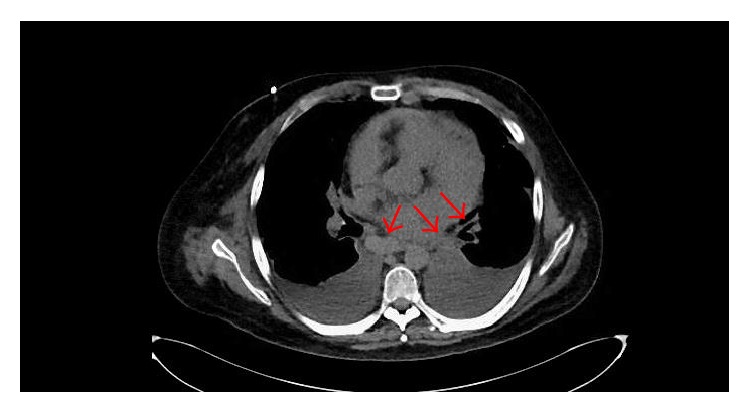
Chest CT with circumferential pericardial and nodular soft tissue densities posterior to the heart as denoted by red arrows.

He was readmitted to the hospital on day 567 after transplant with fever, cough, pleuritic chest pain, and tachycardia. A chest X-ray showed small pleural effusions. He was noted to have EKG changes with T-wave inversions in leads V3–V5 (Figure 1). He had both an elevated troponin at 1.8 ng/mL and an elevated brain natriuretic protein at 805 pg/mL. An echocardiogram demonstrated pericardial effusion and thickened pericardium and could not rule out a restrictive process. A cardiac catheterization showed no significant pathology. A high resolution CT scan of the chest revealed small circumferential pericardial and nodular soft tissue densities posterior to the heart, considered suspicious for metastatic deposits (Figure 2). He ultimately underwent a pericardiectomy.

**Figure 3:**
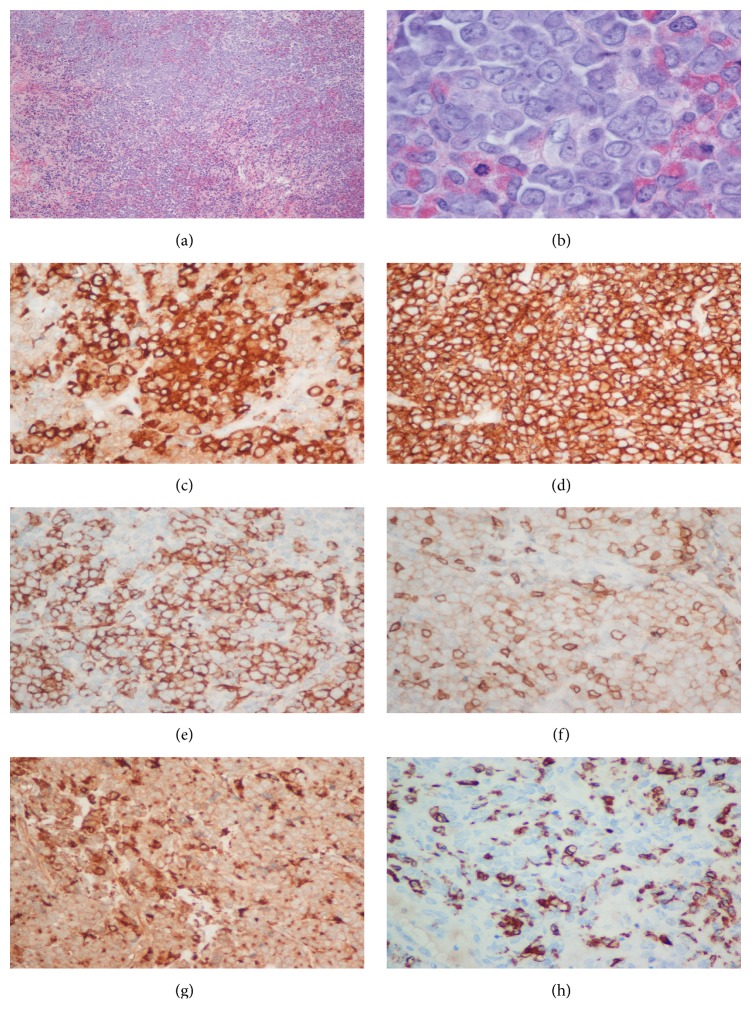
(a) H&E, low power. (b) H&E, high power. (c) Myeloperoxidase focal positivity. (d) CD43 commonly expressed in myeloid sarcoma. (e) CD34 (marker of immaturity). (f) CD117 focal+ (expressed in immature myeloid cells). (g) CD68 weak+ (monocytic/histiocytic markers). (h) CD163 focal+ (monocytic/histiocytic marker).

Histopathological evaluation revealed an infiltrate of atypical cells, seen in sheets and clusters throughout the pericardium and focally within adjoining adipose tissue. By immunohistochemistry, the atypical cells showed an immature myeloid/monocytic immunophenotype: CD34 focal+, CD43+, MPO focal+, CD117 focal+, CD68 weak+, CD163 focal+, PAX5−, CD56−, TdT−, and CD79a focal weak+ (Figure 3).

Chimerism studies of the biopsied tissue revealed that the lesion was entirely donor derived. A bone marrow biopsy showed normal hematopoiesis with full donor chimerism. Thus cardiac myeloid sarcoma represented an isolated site of disease.

Unfortunately, the patient continued to clinically deteriorate. He developed multiorgan failure requiring vasopressor support, hemodialysis, and an intra-aortic balloon pump. He expired on day 579 after transplant.

The umbilical cord blood registry was contacted and the donor was reported to be in good health with no evidence of leukemia.

## 3. Discussion

Donor cell leukemia (DCL) is a rare complication following allogeneic transplants, with an estimated incidence of 0.1% [2]. Acute myeloid leukemia (AML) is the most commonly reported type of DCL, although other neoplasms have been reported, including multiple myeloma, squamous cell carcinoma, and B-cell immunoblastic sarcomas [6]. Isolated donor derived myeloid sarcoma, as developed in our patient, is an extremely rare entity. The diagnosis can be difficult to make particularly in patients with a mixed chimerism following transplant. Reactive donor derived leukocytes can infiltrate a host derived myeloid sarcoma making it difficult to distinguish relapse from a DCL. The use of molecular testing for chimerism, including short tandem repeats (STR) and variable number of tandem repeats (VNTR), has improved the ability to distinguish host from donor derive neoplasms. VNTR was used in our patient to confirm with certainty that the cardiac myeloid sarcoma was donor in origin.

The pathogenesis of DCL remains unknown at this time. The most basic explanation is that the graft harbors occult malignant cells which progress into clinically overt disease after the transplant. In a systematic screening of unselected cord blood samples, approximately 1% of the sampled products harbored small clones positive for TEL-AML1 and 0.2% harbored AML-ETO [7]. However, in the vast majority of patients with DCL, the donor never develops a neoplasm [6]. It is postulated that the host has a compromised immune system and thus lacks the ability to surveille and destroy small neoplastic clones. The immunocompetent donor, by contrast, detects and eliminates these clones without difficulty.

The extreme proliferative demands on transplanted cells following transplantation may further drive this process. The rapidity of proliferation may increase the probability of replication errors and leukemogenicity [7, 8]. Stem cell telomeres undergo accelerated shortening in stem cell transplant recipients [8]. In one study, this telomere shortening was found to be equivalent to 15 years of aging after transplantation, when compared to age matched controls [9]. Shortened telomeres are associated with genomic instability and may favor leukemic transformation.

Another theory suggests that the residual effects of conditioning chemotherapy may damage the graft and cause a therapy related neoplasm as is classically associated with topoisomerase II inhibitors and alkylating agents. In a review by Wiseman, 96% of the patient with DCL had been exposed to either of these two classes of chemotherapeutics during conditioning and 47% had cytogenetic abnormalities consistent with t-AML including −7 and* MLL* gene rearrangements [6, 10, 11].

Not surprisingly, given the comparatively recent introduction of UCBT, fewer cases of DCL leukemia have been reported in the literature when compared with peripheral blood (PBSCT) or bone marrow transplants (BMT). Despite this young history, some interesting trends have emerged. AML predominates as the most common type of UCBT DCL, whereas AML and ALL are similarly represented in DCL following BMT or PBSCT. There also appears to be a shorter interval to the development of DCL in UCBT, with the median time to development of DLC being 14.5 months for UCBT versus 36 months for BMT or PBSCT. Abnormal karyotypes, particularly monosomy 7, are much more common in UCBT DCL than in BMT or PBSCT [12].

Perhaps, UCB is particularly sensitive to residual leukemogenic effects of conditioning chemotherapy. The benefit of time and further research will likely provide more information about the true incidence and mechanism of DCL in UCBT. Given the predominance of AML type DCL in UCBT, it is possible that in the future we may also see more cases of donor myeloid sarcoma, as was the case with our patient. Improved molecular testing, including VNTR, will allow us to detect this entity with greater precision and avoid mistakenly categorizing this phenomenon as relapsed disease.

## Clinical Practice Points

- Donor cell leukemia (DCL) represents a rare complication of allogeneic transplantation.
- The incidence may be higher following cord blood transplants.
- Here, we present an unusual case of a patient with B-cell acute lymphoblastic leukemia (B-ALL) who presented with a donor derived myeloid sarcoma of the heart following a double cord umbilical blood transplant (UCBT).
- Acute myeloid leukemia (AML) is the most common type of UCBT DCL.
- There is a shorter interval to the development of DCL in UCBT compared to bone marrow or peripheral blood stem cell transplantation and abnormal karyotypes are more common in UCBT DCL.
- UCB may be uniquely sensitive to leukemogenic effects of conditioning.
- Improved molecular testing will allow precise detection and avoid mistakenly categorizing DCL as relapsed disease.
